# *ZYG11A* serves as an oncogene in non-small cell lung cancer and influences *CCNE1* expression

**DOI:** 10.18632/oncotarget.6904

**Published:** 2016-01-12

**Authors:** Xin Wang, Qi Sun, Chen Chen, Rong Yin, Xing Huang, Xuan Wang, Run Shi, Lin Xu, Binhui Ren

**Affiliations:** ^1^ Department of Jiangsu Key Laboratory of Molecular and Translational Cancer Research, Cancer Institute of Jiangsu Province, Nanjing, Jiangsu, China; ^2^ Department of The Fourth Clinical College, Nanjing Medical University, Nanjing, Jiangsu, China; ^3^ Department of Cardiothoracic Surgery at Jinling Hospital, Southern Medical University, Nanjing, Jiangsu, China; ^4^ Department of The Second Clinical College, Nanjing Medical University, Nanjing, Jiangsu, China; ^5^ Department of Thoracic Surgery, Jiangsu Cancer Hospital, Nanjing, Jiangsu, China

**Keywords:** ZYG11A, CCNE1, NSCLC, Bioinformatics, Oncogene

## Abstract

By analyzing The Cancer Genome Atlas (TCGA) database, we identified *ZYG11A* as a potential oncogene. We determined the expression of *ZYG11A* in NSCLC tissues and explored its clinical significance. And also evaluated the effects of *ZYG11A* on NSCLC cell proliferation, migration, and invasion both *in vitro* and *in vivo*. Our results show that *ZYG11A* is hyper-expressed in NSCLC tissues compared to adjacent normal tissues, and increased expression of *ZYG11A* is associated with a poor prognosis (HR: 2.489, 95%CI: 1.248-4.963, *p* = 0.010). *ZYG11A* knockdown induces cell cycle arrest and inhibits proliferation, migration, and invasion of NSCLC cells. *ZYG11A* knockdown also results in decreased expression of *CCNE1*. Over-expression of *CCNE1* in cells with *ZYG11A* knockdown restores their oncogenic activities. Our data suggest that *ZYG11A* may serve as a novel oncogene promoting tumorigenicity of NSCLC cells by inducing cell cycle alterations and increasing *CCNE1* expression.

## INTRODUCTION

Lung cancer is the most commonly diagnosed cancer and the leading cause of cancer death worldwide [1], with a 5-year survival rate of less than 15% in most countries, even if patients accept the standard therapies [2, 3]. In recent years, with the development of abundant open data resources. It is now possible for researchers to identify cancer-related genes much more conveniently and effectively [4, 5]. Through an analysis of the TCGA database, we identified a set of 7 novel lung cancer-related candidate genes that were differentially expressed between cancerous and normal tissues. Our previous microarray analysis yielded similar results (with the fold change of 2.46 between lung cancer tissues and normal tissues) [6]. Through a careful review of the literature, we found a number of these genes had been investigated previously. However, among the as yet unknown genes, *ZYG11A* showed a much higher fold-change between cancer and paired normal tissues (with an average 5.88 fold-change in the TCGA-LUNG dataset, *p*<0.00001). We therefore chose *ZYG11A* as the candidate gene for further investigation.

*ZYG11A* belongs to the ZYG11 family of genes, which includes three homologues, *ZYG11A*, *ZYG11B* and *ZER1*, in humans [7]. In *C. elegans*, the orthologous gene *zyg11* is important in meiotic progression and embryonic development [8, 9]. However, the function of this gene in humans remains unknown. In the present study, we demonstrate that *ZYG11A* is a potential oncogene that promotes NSCLC cell proliferation and migration *in vitro* and *in vivo*. Moreover, ectopic expression of *CCNE1* may contribute to the oncogenic function of *ZYG11A*.

## RESULTS

### Bioinformatics analyses implicate *ZYG11A* as a candidate oncogene in NSCLC

**Figure 1:**
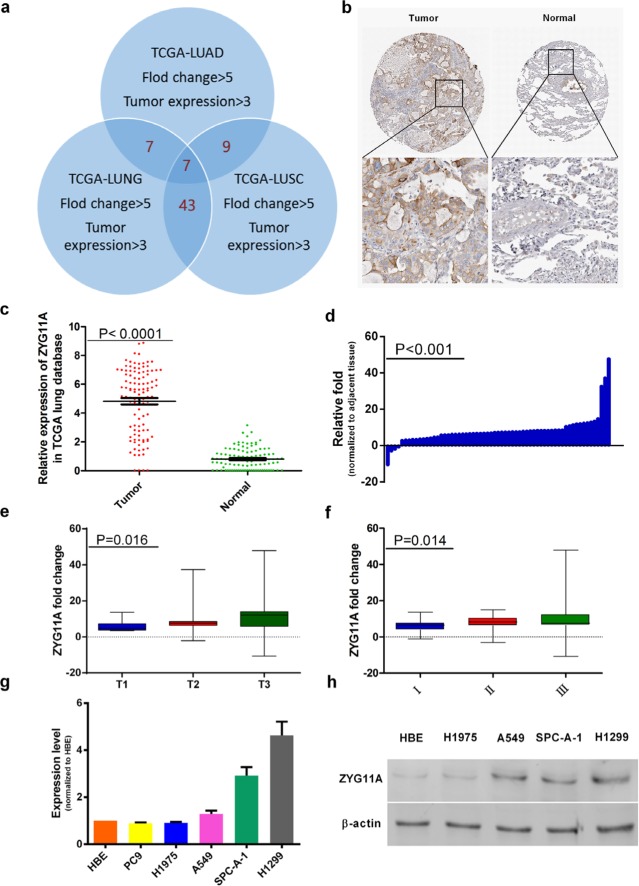
*ZYG11A* is highly expressed in NSCLC tissues **a.** Venn Diagram for gene screening, TCGA datasets genes with fold change > 5 and tumor expression > 3 were included, and a list of 7 genes was obtained. **b.** Normal lung tissues do not express ZYG11A, but several lung tumor tissues are positive for ZYG11A. **c.** After analysis of 108 paired tissues, *ZYG11A* was found to be highly expressed in tumors (*p*<0.0001). **d.** *ZYG11A* is over-expressed in 93.7% (59 of 63) of the lung cancer tissues, with an average over-expression of 9.3-fold (*P*<0.001) as compared to paired normal tissues. **e and f.** ZYG11A over-expression is associated with greater T stage (*p* = 0.016) and TNM stage(*p* = 0.014). **g and h.** ZYG11A mRNA and protein are hyper-expressed in H1299 and SPC-A-1 cell lines.

To identify potential lung cancer-related genes, we first analyzed the TCGA datasets: TCGA_LUNG_exp_HiSeqV2-2015-02-24, TCGA_LUAD_exp_HiSeqV2-2015-02-24, and TCGA_LUSC_exp_HiSeqV2-2015-02-24. For these datasets, genes with a fold change > 5, and tumor expression > 3 were included in our analysis. After intersecting the results from different data sources, we obtained a list of 7 genes (details shown in Supplementary Table 1 and Figure 1a). Then the list was reviewed manually on PubMed and Google Scholar. *PITX2*, *HOXC13*, and *BARX1* act as transcription factors, and have been widely investigated in cancers [10-18]. *DLL3* acts as a Notch ligand that is characterized by a DSL domain, EGF repeats, and has also been studied in cancers [19, 20]. Described by NCBI gene, *LOC100131726* is a long non-coding RNA. *IL1F5*, a member of the interleukin 1 cytokine family, involved in the pathogenesis of psoriasis [21, 22]. Although *ZYG11A* was noted as a cell cycle regulator in the NCBI database, there is little known about the function of *ZYG11A*.

By analyzing the TCGA_LUNG_exp_HiSeqV2-2015-02-24 dataset, compared with normal tissues, *ZYG11A* expression was 5.88-fold hyper-expressed in cancer tissues (*p*<0.0001). When focused on 108 paired tissues (the tumor and paired normal lung tissue from a same patient), *ZYG11A* was over-expressed in tumors compared with paired normal tissues (Figure 1c). Similar results were also observed in lung adenocarcinoma and lung squamous carcinoma databases (Supplementary Figure 1). Then Human Protein Atlas immunohistochemistry (IHC) analyses showed that ZYG11A was not expressed in normal lung tissues, but was expressed in 4 out of 12 (33.3%) NSCLC tumor tissues (Figure 1b).

### *ZYG11A* is over-expressed in NSCLC tumor tissues and correlates with more aggressive clinical characteristics

The expression profile of *ZYG11A* was further validated by qRT-PCR in 63 paired fresh NSCLC patients' tissues (tumor and adjacent normal lung tissues). As shown in Figure 1d, *ZYG11A* was over-expressed in 93.7% (59 of 63) of NSCLC patients, with an average 9.3-fold over-expression (*p*<0.001). Moreover, over-expression of *ZYG11A* was positively correlated with bigger primary tumor size (*p*=0.016) and more advanced TNM stage (*p*=0.014) (Figure 1e, 1f). However, there were no associations between *ZYG11A* expression and age, sex, tumor grade, lymph node metastasis, or cancer type (Table 1).

**Table 1:** Correlation between *ZYG11A* mRNA expression and clinicopathologic characteristic

Characteristics	Numbers of Patients	Percentage	Fold Change	*p*-Value
Age(years)				0.096
<60	19	30.20%	6.1	
>60	44	69.80%	9.9	
Sex				0.269
Male	55	87.30%	9	
Female	8	12.70%	6.8	
Tumor grade				0.752
High	1	1.60%	11.6	
Middle	52	82.50%	8.4	
Low	10	15.90%	10.2	
Lymph node metastasis				0.091
N0	42	66.70%	7.2	
N1	7	11.10%	11.1	
N2	14	22.20%	12	
Primary Tumor				0.016*
T1	14	22.20%	5.9	
T2	36	57.20%	8	
T3	13	20.60%	13.9	
TNM stage				0.014*
I	19	30.20%	6	
II	28	44.40%	8	
III	16	25.40%	13.3	
Cancer Type				0.542
Squamous Carcinoma	13	20.60%	8.5	
Adenocarcinoma	50	79.40%	8.4	

* Significant correlation

### Knockdown of *ZYG11A* inhibits NSCLC cell proliferation, invasion, migration and induces G1 cell cycle arrest *in vitro*

**Figure 2:**
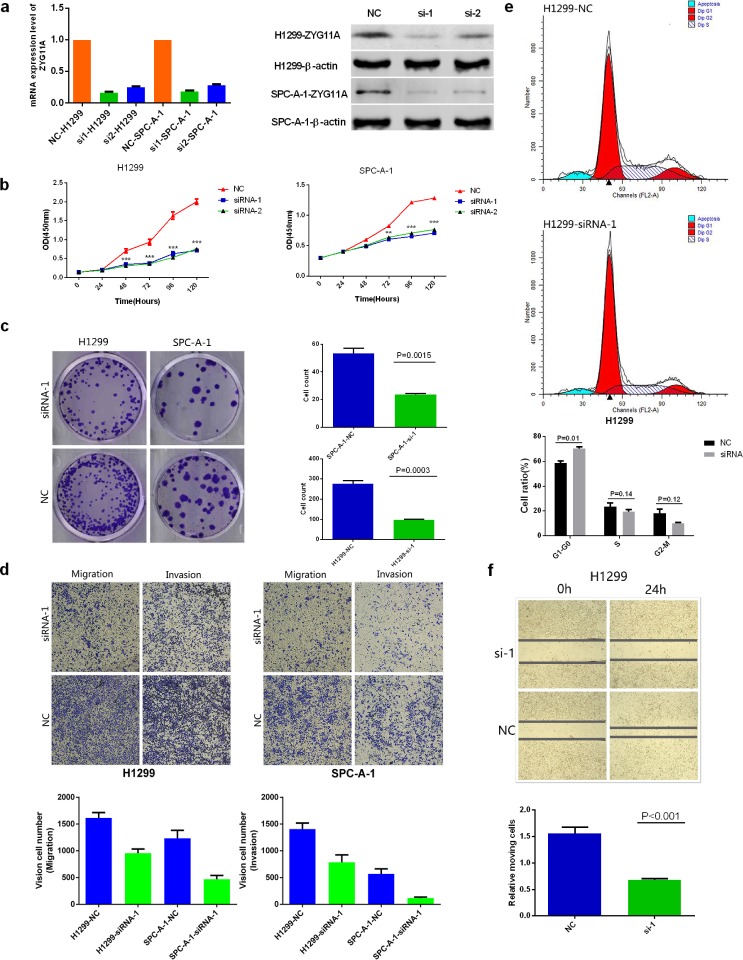
Knockdown of *ZYG11A* alters NSCLC cell line proliferation, migration, invasion, and cell cycle stage *in vitro* **a.** Two specific siRNAs (siRNA-1, siRNA-2) were designed and synthesized, and siRNA-1 had a better efficiency. **b.** Depletion of *ZYG11A* undermines both H1299 and SPC-A-1 cell lines' proliferation **c.** Colony numbers of H1299 and SPC-A-1 cells transfected with si-*ZYG11A* are less than those transfected with si-NC (*p* = 0.0015 and *p* = 0.0003). **d.** Migratory and invasion rates of H1299 or SPC-A-1 cells transfected with si-*ZYG11A* are decreased compared with NC group. **e.** H1299 cells transfected with si-*ZYG11A* display more arrest at G1 phase as compared with cells transfected with si-NC. **f.** si-*ZYG11A* impairs migration as compared with NC group (*p*<0.001).

The expression of *ZYG11A* was compared in different NSCLC cell lines. *ZYG11A* was hyper-expressed in H1299 and SPC-A-1 cell lines as compared with normal human bronchial epithelial (HBE) cells (Figure 1g, 1h). The *Cancer Cell Line Encyclopedia* website also indicated similar results. To investigate the biological function of *ZYG11A in vitro*, two different sets of siRNAs (siRNA-1 and siRNA-2) were utilized to knockdown *ZYG11A*. Both siRNA constructs were able to effectively decrease *ZYG11A* mRNA and protein expression (Figure 2a).

As shown in Figure 2b, cell-counting kit 8 (CCK-8) assays revealed that knockdown of *ZYG11A* reduced proliferation of both H1299 and SPC-A-1 cells. Moreover, si-*ZYG11A* transfected cells had fewer colonies than those transfected with control siRNA (si-NC) (Figure 2c). The trans-well assay showed that migration of H1299 and SPC-A-1 cells was inhibited by siRNA-mediated knockdown of ZYG11A (Figure 2d), and the wound healing assay yielded similar results (Figure 2f). The matrigel invasion assay also revealed that si-*ZYG11A* treatment impaired the invasion capacities of H1299 and SPC-A-1 cells (Figure 2d). Finally, the effect of *ZYG11A* on cell cycle distribution and apoptosis was evaluated by flow cytometry analysis. As shown in Figure 2e, si-ZYG11A treatment increased the percentage of H1299 cells in G1 phase compared to si-NC. However, there was no difference in apoptosis between groups (Supplementary Figure 2).

### Knockdown of *ZYG11A* suppresses tumor growth *in vivo*

**Figure 3:**
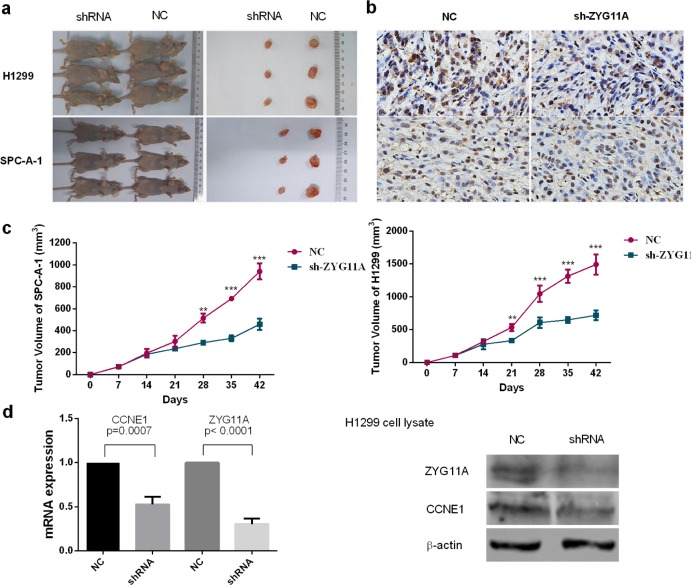
Knockdown of *ZYG11A* retards tumor growth *in vivo* **a.** Tumor nodules from mice injected with sh-*ZYG11A* cells are significantly smaller than those injected with NC cells. **b.** sh-*ZYG11A* tumors from both H1299 and SPC-A-1 cell lines have less dense PCNA staining. **c.** Compared with the NC group, the sh-*ZYG11A* group has reduced tumor size. **d.** Both mRNA and protein expression of CCNE1 are decreased in the sh-*ZYG11A* group compared with NC group, and *ZYG11A* expression is also decreased.

We next used a nude mouse xenograft assay with H1299 and SPC-A-1 cells. When compared with the control group, tumor volumes were smaller in the sh-*ZYG11A* treated groups of both cell lines (Figure 3a, 3c). Xenografts were immunohistologically stained for proliferating cell nuclear antigen (PCNA). Compared with controls, the sh-*ZYG11A* group showed less PCNA staining (Figure 3b), suggesting that *ZYG11A* knockdown could inhibit tumor growth *in vivo*. Intriguingly, expression of *CCNE1* was also decreased in the sh-*ZYG11A* group (Figure 3d).

### *ZYG11A* exerts its oncogenic activity via promoting *CCNE1* expression

**Figure 4:**
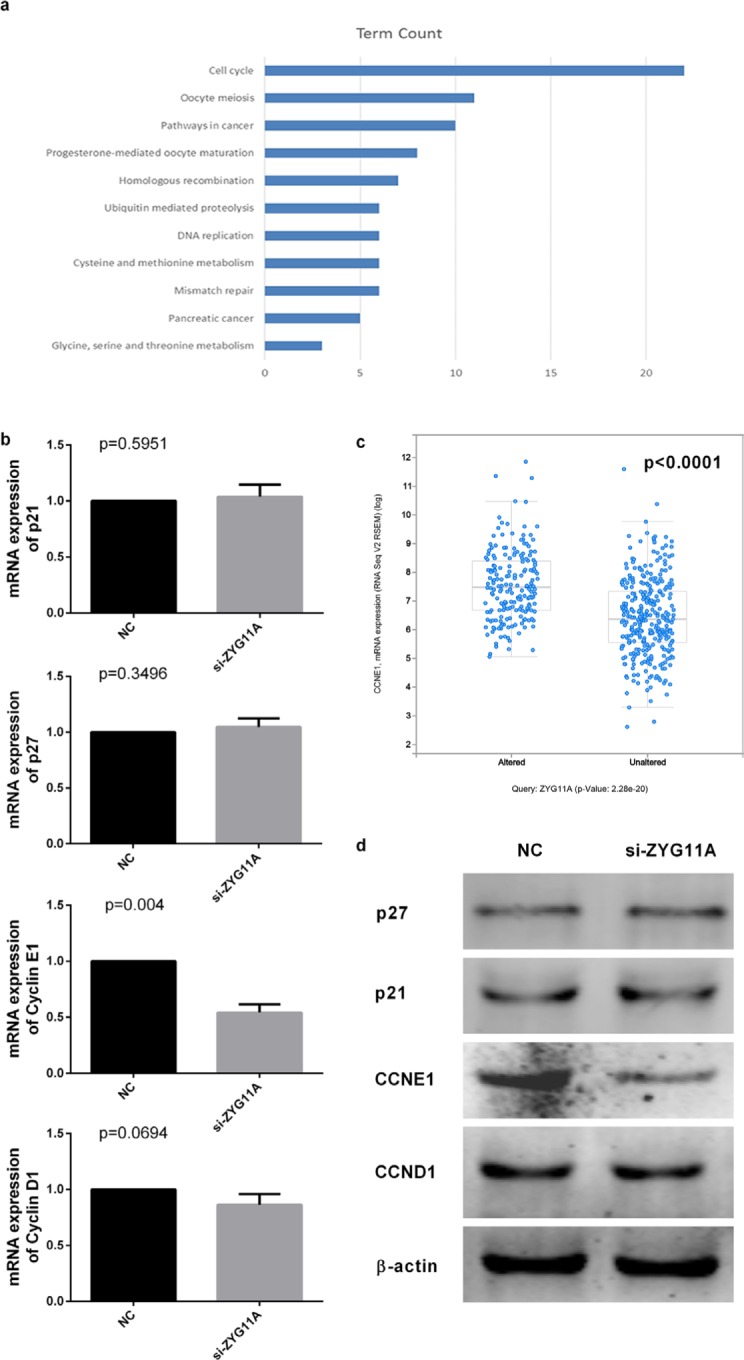
*ZYG11A* depletion influences *CCNE1* expression **a.** KEGG pathway enrichment analysis indicates genes co-expressed with *ZYG11A* are enriched for cell cycle pathways. **b.** *CCNE1* mRNA expression is reduced after transfection with si-*ZYG11A* (*p* = 0.004), but the expression of *CDKN1 (p21)*, *CDKN2 (p27)*, and CCND1 were not altered. **c.** cBioPortal enrichment analysis indicated *CCNE1* expression is positively related with *ZYG11A* expression (*p*<0.001). **d.** CCNE1 protein expression is decreased after transfection with si-*ZYG11A*, with no difference in expression of p21, p27, or CCND1.

**Figure 5:**
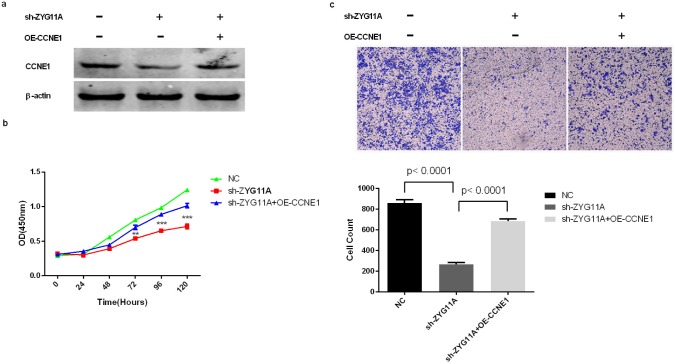
Rescue of proliferation and migration by over-expression of CCNE*1* in *ZYG11A*-depleted cells **a.** *ZYG11A*-depleted H1299 cells transfected with a full-length human *CCNE1* have increased expression of *CCNE1*. **b.** Depletion of *ZYG11A* undermines H1299 cell proliferation, but over-expressing *CCNE1* recovers proliferation. **c.** H1299 cells transfected with sh-*ZYG11A* have a decreased migratory rate when compared with NC transfected cells, but *CCNE1* over-expression restores migration.

We next used KEGG pathway analysis (DAVID Bioinformatics Resources 6.7) on a list of genes co-expressed with *ZYG11A* that was obtained from cBioPortal using both RNA-seq and microarray results of Lung Adenocarcinoma (TCGA, Provisional). Most of the genes co-expressed with *ZYG11A* were enriched in the cell cycle pathway (Figure 4a and Supplementary Table 2). Enrichment analysis using cBioPortal revealed that *CCNE1* expression was positively correlated with *ZYG11A* expression (*p*<0.001) (Figure 4c), but not *CDKN1A*, *CDKN1B*, or *CCND1* (Supplementary Figure 3). Given our findings that *ZYG11A* knockdown decreased cancer cell proliferation, migration, and invasion and promoted G1 cell cycle arrest, we sought to determine expression of cell cycle-related genes in *ZYG11A* knockdown cells using qRT-PCR and western blot analyses. Consistent with enrichment analyses, when compared with si-NC treatment, si-*ZYG11A* treatment decreased both mRNA and protein levels of *CCNE1*, whereas expression of *CDKN1A (p21)*, *CDKN1B (p27)*, and *CCND1* were not influenced (Figure 4b, 4d). Then *CCNE1* was ectopically expressed in *ZYG11A* knockdown H1299 cells (Figure 5a), cell proliferation and invasion were recovered, as determined by the CCK8 and transwell assays (Figure 5b, 5c).

### Prognostic value of ZYG11A protein expression in lung cancer patients

**Figure 6:**
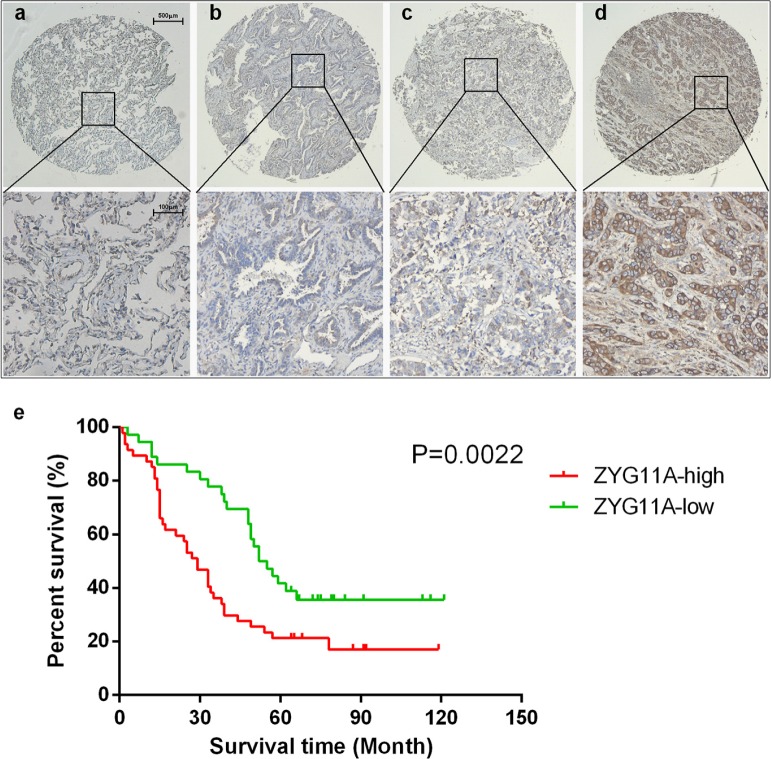
Tissue microarray analysis **a, b, c, d.** each present an example of normal, I, II, and III TNM stage, ZYG11A expression is associated with TNM stage. **e.** univariate survival analysis indicated that ZYG11A expression is associated with poorer prognosis *p* = 0.0022.

ZYG11A protein was also increased in tumor tissues. After excluding 8/90 tissue pairs for missing data/dots (7 tumor tissues and 1 normal tissues), expression of *ZYG11A* protein was higher in tumor tissues compared with their relative normal tissues (in 67/82 tissues examined). High *ZYG11A* expression (as determined by a cut-off score of 140) was detected in 47 (56.6%) of the 83 lung cancer tissues, compared with only 12 (13.5%) of 89 adjacent normal tissue samples. There was a positive correlation between *ZYG11A* protein expression and patients' TNM stage (Figure 6a, 6b, 6c, 6d Table 2). Multivariate analyses indicated that increased expression of *ZYG11A* was associated with poorer overall survival rate (HR: 2.489, 95%CI: 1.248-4.963, *p* = 0.010). Greater T stage and TNM stage were also associated with poorer prognosis (Table 3). Kaplan-Meier survival curves are shown in Figure 6b (*p* =0.0022).

**Table 2:** Sequences of qRT-PCR primer

Gene	sense	anti-sense
*ZYG11A*	CCCTCCTGACGCTCAGAAG	AGATGTTGACCAAAGTGTAGGGA
*CDKN1B*	TGGAGAAGCACTGCAGAGAC	GCGTGTCCTCAGAGTTAGCC
*CDKN1A*	GCAGACCAGCATGACAGATTT	GGATTAGGGCTTCCTCTTGGA
*CCND1*	GCGCTTCCAACCCACCCTCCATG	GCGCCGCAGGCTTGACTCCAGAA
*CCNE1*	TTCTTGAGCAACACCCTCTTCTGCAGCC	TCGCCATATACCGGTCAAAGAAATCTTGTGCC
*ACTB*	GAAATCGTGCGTGACATTAA	AAGGAAGGCTGGAAGAGTG

**Table 3:** Correlation between ZYG11A protein expression and clinicopathologic characteristic

Groups		ZYG11A		Pearson χ2	*P-value*
		High expression	Low expression		
Gender				0.17	0.68
	Male	24	20		
	Female	23	16		
Age				0.11	0.74
	<60	20	14		
	≥60	27	22		
Differentiation				0.09	0.76
	I-II	35	31		
	III-IV	3	2		
T stage				2.51	0.11
	T1-T2	32	30		
	T3-T4	15	6		
Lymph node metastasis				0.03	0.86
	negative	22	16		
	positive	18	12		
TNM stage				3.99	0.04*
	I-II	10	23		
	III-IV	19	16		

* Significant correlation

## DISCUSSION

*ZYG11A* is a member of the *ZYG11* gene family and was originally cloned by Pawlak et al. and defined as a potential cell cycle regulator [23]. Subsequent studies revealed that the *ZYG11* family involved in cell division during meiosis[24]. Its homologue ZYG11B was reported serve as a substrate recruitment subunit for a cullin-2-based E3 ubiquitin ligase [8, 9]. Dysregulation of cullin-2-based E3 ubiquitin system is associated with numerous human diseases, including cancer [23, 25], and correlated with the prognoses of cancer patients [26, 27].

In this study, we present evidence that *ZYG11A* is over-expressed in NSCLC and that over-expression of *ZYG11A* is associated with greater tumor size and more advanced TNM stage. *ZYG11A* is also hyper-expressed in several NSCLC cell lines as compared to a normal HBE line. Using CCK8 assays, apoptosis analyses and colony formation assays, we determined that *ZYG11A* knockdown inhibits cell proliferation and tumorigenesis, with no effect on apoptosis. Transwell and Matrigel assays further revealed that *ZYG11A* knockdown undermines cancer cell migration and invasion abilities. Xenograft assays showed that depletion of *ZYG11A* suppressed proliferation of cancer cells *in vivo*, while flow cytometric analysis indicated that si-*ZYG11A* treatment strongly inhibits G1 cell cycle progression, without increased apoptosis.

KEGG and cBioPortal enrichment analyses yielded similar results. We therefore hypothesize that *ZYG11A* promotes proliferation, migration and invasion of NSCLC cells by inducing G1 cell cycle progression. As previously mentioned, the *ZYG11* family may work with E3 ubiquitin ligases to influence cell cycle progression [8, 9, 23, 24, 28, 29]. We therefore measured expression of several cell cycle-related genes to explore the potential mechanisms underlying the oncogenic activity of *ZYG11A*. We found that *CCNE1* expression is specifically decreased by siRNA-mediated *ZYG11A* knockdown, which was consistent with cBioPortal enrichment analysis (*p*<0.001).

Cell cycle alteration is one of the hallmarks of cancer [30-32]. *CCNE1* is a classic G1/S cell cycle-related protein [33, 34]. Therefore, rescue experiments were performed. We found that ectopic expression of *CCNE1* in H1299 cells with *ZYG11A* knockdown greatly increased their proliferation and migration abilities. Finally, we explored the prognosis values of *ZYG11A*. Both univariate and multivariate analysis showed that lung cancer patients strongly expressing ZYG11A have a poorer prognosis than those with absent or weaker ZYG11A expression.

Our study suggests that *ZYG11A* is hyper-expressed in NSCLC and correlates with larger primary tumor size and more advanced TNM stage. *ZYG11A* can promote proliferation, migration, and invasion of NSCLC cells *in vitro* and accelerate tumor growth *in vivo*. In addition, *ZYG11A* depletion suppresses cell cycle progression by inhibiting *CCNE1* expression. This suggests *ZYG11A* is an oncogene in NSCLC and may represent a novel diagnostic and therapeutic target for treatment of NSCLC.

## MATERIALS AND METHODS

### Data sources and bioinformatics

Level 3 TCGA data: *TCGA_LUNG_exp_HiSeq V2-2015-02-24, TCGA_LUAD_exp_HiSeqV2-2015-02-24* and *TCGA_LUSC_exp_HiSeqV2-2015-02-24* were downloaded at the website of the UCSC cancer browser (http://genome-cancer.ucsc.edu), containing 109, 58, and 51 paired normal lung tissue samples respectively. All mRNA expression values were normalized, and values for *ZYG11A* expression were obtained from the “*genomicMatrix*” file (using Editplus® software). Fisher's t-test was used to compare the two groups. In order to determine the protein expression level ZYG11A, the Human Protein Atlas (http://www.proteinatlas.org) was used following their guidelines [35-38]. Then, in order to pick an appropriate cell line for functional research, the Cancer Cell Line Encyclopedia (http://www.broadinstitute.org/ccle/home) was used [39], and the expression of *ZYG11A* in NSCLC cells was evaluated. Finally, we retrieved and analyzed the data of TCGA using the co-expression and enrichment analysis tool in cBioPortal (http://www.cbioportal.org/) [40]. The list of all genes with highest expression correlation with *ZYG11A* was submitted to DAVID Bioinformatics Resources 6.7 (http://david.abcc.ncifcrf.gov) for KEGG pathway enrichment analysis as previously described [4, 41-43].

### Tissue samples and animal studies

A total of 63 patients had undergone curative surgical resection at Nanjing Medical University Affiliated Cancer Hospital from 2010 to 2013. Two pathologists performed the histopathological classifications in a double-blind fashion. No patient had received preoperative chemotherapy or radiotherapy. The clinical and pathological characteristics of the patients aged between 42 to 84 years (mean: 63) are summarized in Table 1.

For IHC assay based on a TMA (tissue microarray), 180 formalin-fixed paraffin-embedded (FFPE) paired tissue samples from 90 patients were used (after excluding missing data/dots, 89 normal lung tissues, 83 tumor tissues, and 82 tumor/normal pairs were included in further analysis). These tissues were obtained from the Shanghai Biochip Co., Ltd., Shanghai, China (Cat. No. HLug-Ade180Sur-01). Operations occurred between July 2004 and June 2009, All tissues were re-examined by an experienced pathologist after they were transferred from a local hospital and the TNM stage was determined in each patient. This research protocol was approved by the Human Research Ethics Committee of Nanjing Medical University.

All animal studies were conducted in accordance with NIH animal use guidelines and protocols approved by Nanjing Medical University Animal Care Committee. Twelve female nude mice (ages 4–6 weeks) were purchased from Nanjing Medical University School of Medicine's accredited animal facility. Briefly, 1.0×10^6^ exponentially growing cells with ectopic expression of appropriate genes were injected subcutaneously. Tumor volume was estimated using calipers every week ((length* width^2)/2) [44], then in the sixth week after injection, animals were sacrificed. Tumor nodules were harvested and measured, then immunohistochemically stained with PCNA to assess the proliferation of cells.

### Cell lines, cell culture, siRNA, and Lentivirus-based RNA interference transfection

H1299 and A549 cells were obtained from American Type Culture Collection (ATCC, USA), PC-9 cells were a generous gifted from Dr. Zhian Liu, while human bronchial epithelial cell (HBE) and SPC-A-1 cells were gifted by Dr. Zhibin Hu. All cells were grown in RPMI1640 media (Kaiji, Nanjing, China) supplemented with 10% fetal bovine serum and penicillin/streptomycin and cultured at 37°C in a humidified incubator containing 5% CO_2_. Transfection was performed following the small-interfering RNA (siRNA) sequences transfection protocol for Lipofectamine RNAi MAX (Invitrogen, USA). Nonsense RNAi (nsRNA) was used as a negative control for *ZYG11A* siRNA. Transfection efficiency was evaluated by quantitative real-time RT-PCR and western blot. Two siRNAs were designed; the sequences were as follows: siRNA-1 for *ZYG11A*: sense 5′-GCAGUCAUUAGAGAACUUATT-3′, antisense 5′-UAAGUUCUCUAAUGACUGCTT-3′; siRNA-2 for *ZYG11A*: sense 5′-CCAGUUCCAGACAUCAUAATT-3′, antisense 5′-UUAUGAUGUCUGGAACUGGTT-3′. And the following Nonsense siRNA was used as control: sense 5′- UUCUCCGAACGUGUCACGUTT-3′, antisense 5′-ACGUGACACGUUCGGAGAATT-3′. The human *ZYG11A* targeting small hairpin RNA sequence was designed based on siRNA-1 and a negative control sequence 5′-GCACTACCAGAGCTAACTCAGATAGTACT-3′. We generated recombinant lentiviral particles and cells were transfected with *ZYG11A* or negative control recombinant lentivirus (sh-*ZYG11A* or sh-NC, respectively) as described in our previous article [45]. A Genechem-CCNE1 plasmid expressing full-length human *CCNE1* was purchased from Genechem, and an empty plasmid was used as a negative control.

### RNA preparation, reverse transcription, and real-time quantitative PCR

Total RNA was extracted from tissues or cultured cells using TRIzol reagent (Invitrogen, Carlsbad, CA, USA). For RT-PCR, 1000 ng total RNA was reverse-transcribed to a final volume of 20μl cDNA using a Reverse Transcription Kit (Takara, cat: RR036A). qRT-PCR analyses were performed with SYBR Select Master Mix (Applied Biosystems, Cat: 4472908). The qRT-PCR primers for *ZYG11A*, *CDKN1A*, *CDKN1B*, *CCND1*, *CCNE1*, and *ACTB* are shown in Table 4. The qRT-PCR data collection was performed using a QuantStudio™ 6 Flex Real-Time PCR System and the qRT-PCR reaction included an initial denaturation step at 95°C for 10 min, followed by 40 cycles of 92°C for 15 sec and 60°C for 1 min. Each sample was run in triplicate and the relative expression of *ZYG11A* was calculated and normalized using the 2^−ΔΔCt^ method relative to *ACTB*.

**Table 4:** Analysis of independent correlation factors of lung cancer prognosis with Cox multivariate regression analysis

Factor	SE	Wald	DF	*P-value*	HR	95%CI	
						Lower	Upper
Gender (male vs female)	0.419	0.007	1	0.933	0.965	0.425	2.196
Age (≥60 vs <60)	0.402	0.417	1	0.518	1.297	0.590	2.851
Differentiation (III-IV vs I-II)	0.734	0.05	1	0.824	1.178	0.279	4.963
T stage (T1-T2 vs T3-T4)	0.535	4.055	1	0.044*	0.340	0.119	0.972
Lymphatic metastasis (positive vs negative)	0.520	1.443	1	0.230	1.867	0.674	5.170
TNM stage (III-IV vs I-II)	0.611	5.863	1	0.015*	4.386	1.325	14.515
*ZYG11A* expression (high vs low)	0.352	6.708	1	0.010*	2.489	1.248	4.963

SE: standard error; DF: degree of freedom; HR: hazard ratio; CI: confidence interval; Lower: lower limit; Upper: upper limit.

* Significant correlation

### Protein preparation and western blot

Whole cells were homogenized and treated with lysis buffer on ice (Kaiji, Nanjing, China), and a BCA kit (Kaiji, Nanjing, China) was used to quantify protein concentrations. Equal amounts of protein were loaded in SDS–PAGE gels. After separation in the gel, the protein was transferred onto a PVDF membrane. The membranes were blocked in 2% BSA in TBST for 1 h, and incubated overnight (4°C) with antibodies against ZYG11A (Abcam, ab177696 1:1000), p21 (santa cruz, sc-397 1:500), p27 (santa cruz, sc-528 1:200), Cyclin D1 (CST, 2978 1:1000), Cyclin E1 (abcam, ab7959 1:200) or beta-actin (Cell Signaling, 8H10D10 1:1000). After washing in TBST, the membrane was incubated with goat anti-rabbit HRP-conjugated secondary antibody (1:10,000; Abcam) or goat anti-mouse HRP-conjugated secondary antibody (1:10,000; Abcam) for 2 h at room temperature. The blots were visualized by ECL detection (Thermo Scientific), and all experiments were repeated triple times.

### Immunohistochemistry

Tissue sections were deparaffinized and rehydrated through graded alcohol. Endogenous peroxidase activity was blocked by incubation in 3% H_2_O_2_. Antigen retrieval was carried out with 0.01 M citrate buffer (pH 6.0) and microwave heat induction. An anti-ZYG11A rabbit polyclonal antibody (CST, HPA030378 1:300) was used.

ZYG11A staining was scored by blinded observers (including a pathologist) according to intensity and percentage of positive cells. The staining intensity was scored according to 4 grades: 0 (No staining), 1 (weak staining), 2 (moderate staining), or 3 (intense staining). The product (percentage of positive cells and respective intensity scores) was used as the final staining score (a minimum value of 0 and a maximum of 300). 140 was used as a cut-off point score and is statistically significant as determined by X-tile software (the Rimm Lab at Yale University; http://www.tissuearray.org/rimmlab/) [46].

### Cell proliferation assay

The cell proliferation rate was measured using a Cell Counting Kit-8 (Kaiji, Nanjing, China). Cells were plated in 96-well plates at a density of 2000 cells in 100ul per well and the absorbance was measured at 450 nm with an ELx-800 Universal Microplate Reader. Each experiment was repeated quadruplicate at various time points for 6 days.

### Clonogenic assay

For colony formation assays, a total of 100 transfected cells were placed in a fresh six-well plate and maintained in media containing 10% FBS, replacing medium every 3 or 4 days. After two weeks, cells were fixed with 4% paraformaldehyde and stained with 0.1% crystal violet. Visible colonies were manually counted; and each experiment was repeated three times.

### Cell invasion, migration, and wound healing assay

For migration assays, transfected cells (40,000 cells in 100ul per well) were plated in the upper chamber of trans-well assay inserts (8 mm pores, Millipore, Billerica, MA) containing 200ul of serum-free RPMI1640 media. The lower chambers were filled with RPMI1640 containing 10% FBS. After 24 h of incubation, cells on the filter surface were fixed with methanol, stained with crystal violet, and photographed. Migration was assessed by counting the number of stained cell nuclei from 5 random fields per filter in each group.

For invasion assays, transfected cells (40,000 cells in 100ul per well) were plated in the top chamber with a matrigel-coated membrane (BD Biosciences) in 300ul serum-free RPMI1640. The bottom chambers were filled with RPMI1640 containing 10% FBS. Invasion was determined after 48 h incubation.

Wound healing assay, cells were seeded and transfected on six-well plates with si-ZYG11A or si-NC, then an artificial scratch wound on a confluent monolayer of H1299 cells was created with a 200-μl pipette tip. Serum-free medium was added for a further 24-h, and cells were imaged 24 h later. Each experiment was repeated three times.

### Flow-cytometry analysis

Flow-cytometry analysis was performed detecting cell cycle distribution and cell apoptosis. For cell cycle distribution, cells were transferred and fixed in centrifuge tubes containing 4.5 mL of 70% ethanol on ice. The cells were kept in ethanol for at least 2 h at 4°C, then centrifuged for 5 min at 300g. Cell pellets were resuspended in 5 mL of PBS for approximately 30s and centrifuged at 300 g for 5 min, then resuspended in 1 mL of PI staining solution and kept in the dark at 37°C for 10 min. Samples were analyzed using a FACSCalibur flow cytometer. The percentage of the cells in G0–G1, S, and G2–M phase were counted and compared. For apoptosis analysis, briefly, cells were washed and resuspended at a concentration of 1 × 10^6^ cells/ml. Then an Annexin V-FITC Apoptosis Detection Kit (BD Biosciences) was used following the manufacturer's protocol. After incubation at room temperature in the dark for 20 min, the cells were immediately analyzed by a FACScan flow cytometer (Becton Dickinson, Franklin Lakes, NJ). Each assay was performed in triplicate.

### Statistical analysis

Data are presented as means ± S.D. and statistical analysis was performed using Student's *t* test or one-way ANOVA, Cox multivariate regression, or Kaplan-Meier survival analysis (SPSS Statistics, version 20.0, Chicago, Ill). *P*<0.05 were considered statistically significant. The data graphs were made with GraphPad Prism 6.0 software.

## SUPPLEMENTARY FIGURES AND TABLES






