# Supplementary material for: ZYG11A serves as an oncogene in non-small cell lung cancer and influences CCNE1 expression
Source: Oncotarget. 2016 Jan 12;7(7):8029–42. doi: 10.18632/oncotarget.6904 (PMC4884973; doi:10.18632/oncotarget.6904)
Supplement: Supplementary file 1 [file oncotarget-07-8029-s001.pdf]

SUPPLEMENTARY FIGURES

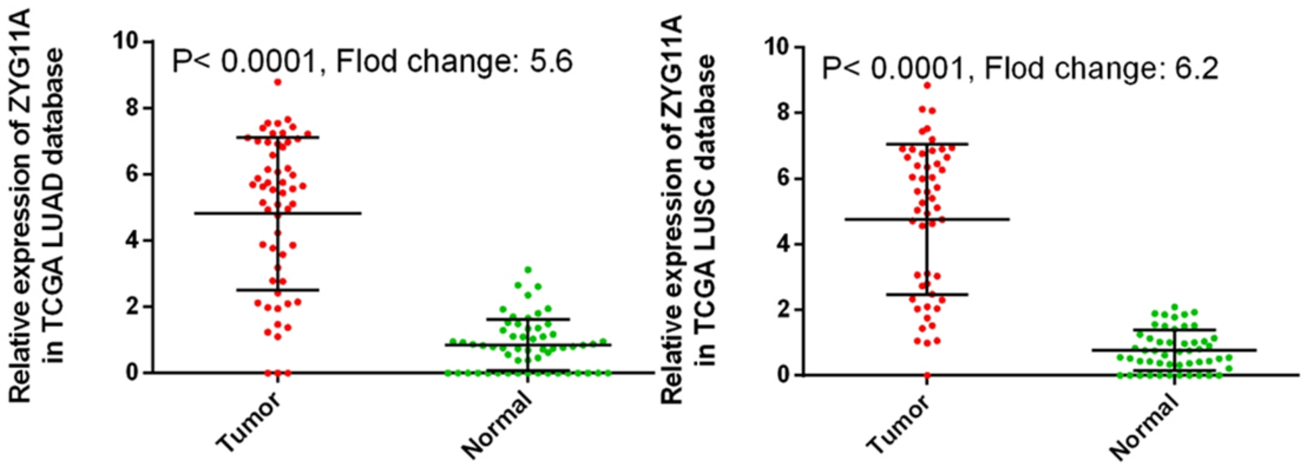

Supplementary Figure S1: TCGA_LUAD_exp_HiSeqV2-2014-08-28 and TCGA_LUSC_exp_HiSeqV2-2015-02-24 were used, indicating *ZYG11A* is highly expressed in both squamous cell lung carcinoma and lung adenocarcinoma.

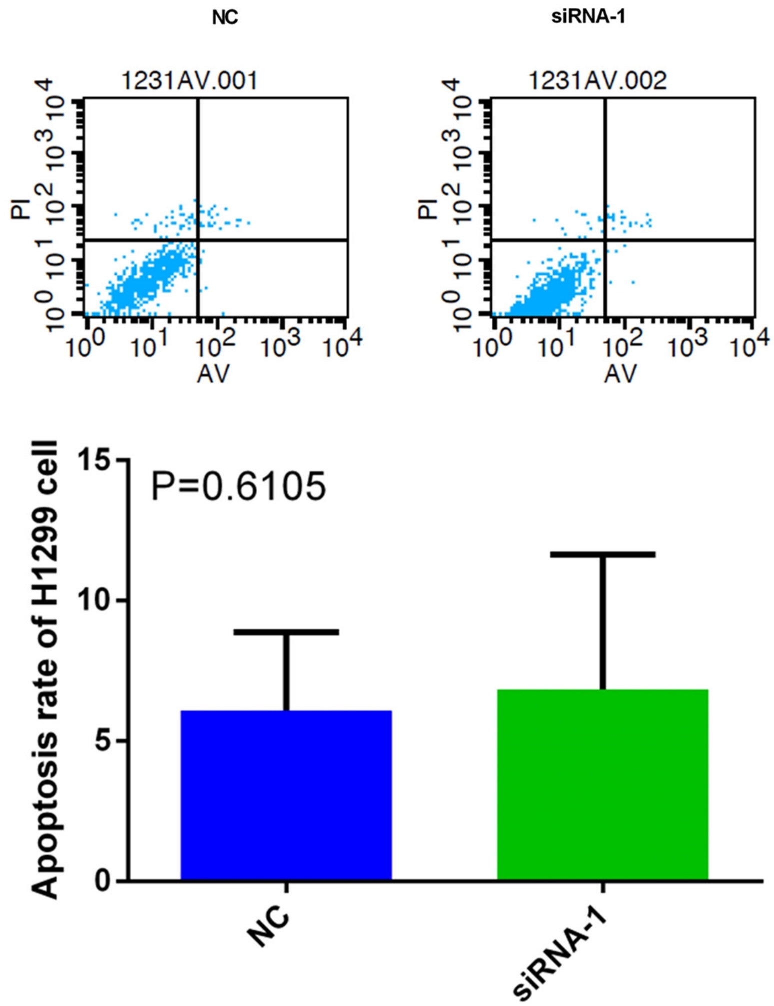

Supplementary Figure S2: In apoptosis analysis no differences were observed between the si-*ZYG11A* group and si-NC group.

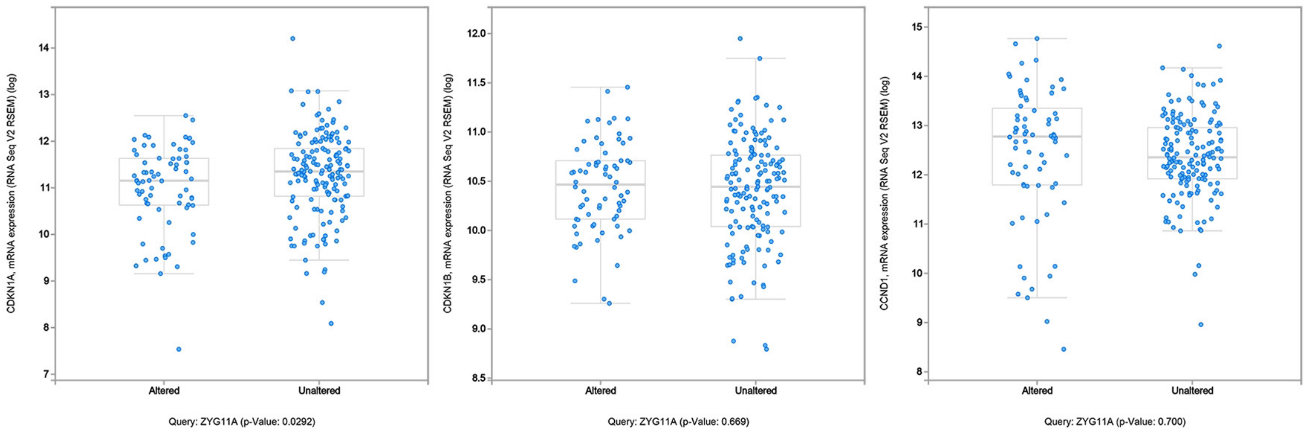

Supplementary Figure S3: In cBioPortal enrichment analysis, CDKN1A (p21) expression was related with ZYG11A expression, but not CDKN1B (p27) or CCND1 expression.

Supplementary Table S1: The identification of potential lung cancer-related genes

See Supplementary File 1

Supplementary Table S2: The list of genes co-expressed with ZYG11A

See Supplementary File 2
